# The Effect of Perceived Social Support on the Mental Health of Homosexuals: The Mediating Role of Self-Efficacy

**DOI:** 10.3390/ijerph192315524

**Published:** 2022-11-23

**Authors:** Weigang Pan, Yujie Zhao, Yihong Long, Ying Wang, Yingzhi Ma

**Affiliations:** 1 Laboratory of Emotion and Mental Health, Chongqing University of Arts and Sciences, Chongqing 402160, China; 2 School of Public Administration, South China University of Technology, Guangzhou 510641, China; 3 College of National Culture and Cognitive Science, Guizhou Minzu University, Guiyang 550025, China

**Keywords:** homosexuals, perceived social support, self-efficacy, mental health, mediating effects

## Abstract

Objective: The purpose of this study was to investigate the mental health of homosexual adults in China and to explore the effects of perceived social support and self-efficacy on mental health. Methods: Two hundred and nine homosexuals were recruited to participate in the online survey. The Perceived Social Support Scale, General Self-efficacy Scale, Self-rating Anxiety Scale and Self-rating Depression Scale were completed through a questionnaire website. Correlation analysis and mediation analysis were used to investigate the relationship between perceived social support, self-efficacy and mental health of homosexuals. Results: Description analysis showed the following: (1) In this study, 53.9% of the participants had depression symptoms and 46.7% had anxiety problems; (2) perceived social support, self-efficacy, anxiety and depression were significantly related to each other. The mediation effect analysis found that self-efficacy mediates between perceived social support and depression but does not mediate between perceived social support and anxiety. Conclusions: The results demonstrated that in the context of Chinese collectivist culture, homosexuals have certain mental health problems, and their perceived social support and self-efficacy are critical protective factors for mental health. Our findings highlight the need to further strengthen social support and self-efficacy in mental health services for Chinese homosexuals.

## 1. Introduction

### 1.1. Challenges Faced by Homosexuals

Since homosexuality was removed from the category of mental disorders in the Diagnostic and Statistical Manual of Mental Disorders, the mental health of sexual minorities has attracted extensive attention in sociology, psychology, public health, epidemiology and other fields [1,2]. According to the minority stress theory, homosexuals face not only external pressures such as prejudice and discrimination from society, but also internal pressures such as self-identity and fear of rejection [3]. Studies show that homosexuals experience higher rates of poor mental health outcomes compared to heterosexual individuals [4,5]. Specifically, depression and anxiety are typical symptoms reported frequently by homosexuals [6]. A longitudinal study that followed sexual minorities for eight years showed that their depressive symptoms did not diminish over time and that social anxiety even increased over time [7]. In addition to internalizing mental disorders such as depression and anxiety, homosexuals are often at risk for externalizing behavioral disorders [8]. A structured diagnostic interview study of urban sexual minority males found that more than half of the individuals had symptoms such as behavioral disorders, alcohol abuse or dependence and nicotine dependence [9].

In different cultural backgrounds, homosexuals may face different sources of psychological pressure. Minority stress theory has been widely applied in western culture [10]. However, the psychological stressors of homosexuals in China, a country with a predominantly Confucian culture, are different from those in the West. A study examined Chinese university students’ attitudes toward same-sex attraction and behavior and found that they indicated generally negative attitudes toward same-sex attraction and behavior, with males reporting more negative attitudes than females [11]. Prejudice, stigma, discrimination and social exclusion against homosexuals still persist in Chinese society [4,5,12]. A number of studies have consistently confirmed that Chinese homosexuals demonstrated a high prevalence of internalized homophobia [13]. Due to the influence of traditional Chinese Confucian culture, the idea of getting married and having children and carrying on the family line is deeply rooted in the hearts of the older generation [4,13,14]. Researchers found that participants who identified with traditional Chinese values of filial piety and who believed their parents were more traditional were more negative about their homosexuality [15]. Therefore, unlike in the West, homosexuals in China face greater pressure and are vulnerable to psychological distress. Accordingly, exploring the mental health status of homosexuals in China and the key protective factors affecting mental health can provide a theoretical basis for the effective development of targeted mental health services.

### 1.2. Protective Factors of Mental Health

In a collectivist culture, the Chinese self is other-oriented [16], and interpersonal relationships play a key role in improving mental health [17] and subjective well-being [18]. Perceived social support is an individual’s subjective perception of the various kinds of help he or she receives from family, friends and significant others [19]. It has been found that perceived social support has a significant predictive effect on individuals’ anxiety and depression symptoms [20]. Among college students, the higher the quality of social support, the lower the likelihood of depression, anxiety, suicide and eating disorder symptoms [21]. Parents who lost their only child experience poor mental health, and social support is expected to alleviate mental stress and facilitate mental adaptation of bereaved parents [22]. For homosexuals, social support has been found to be an important protective factor for mental health [23]. There are two hypotheses about the influence mechanism of social support on mental health: the main effect model and the buffer model [24]. In the main effect model, social support is preventative and has a universally beneficial effect on the physical and mental health of individuals; in contrast, the buffer model proposed that social support can buffer the negative effects of stressful events on physical and mental health [25].

The pressure homosexuals faced in their life mainly comes from the discrimination and prejudice against their sexual orientation from the outside world, whereas social support of their sexual orientation can alleviate their psychological problems [26]. A recent study found that a very important way of improving the anxiety of homosexuals is by enhancing their parents’ understanding and acceptance of homosexuality and making them dare to seek support from their parents [27]. In addition, social support from friends has a positive effect on improving young gays’ life satisfaction [28]. These results suggest that support from family and friends may improve the mental health of homosexuals. Therefore, this study aims to verify the relationship between perceived social support and mental health status represented by anxiety and depression in homosexuals and proposes Hypothesis 1: Perceived social support of homosexuals may negatively predict mental health status, with depression and anxiety as indicators.

In addition to social support, another positive psychological resource for coping with stress is self-efficacy. Self-efficacy refers to people’s belief that they can successfully complete their work and solve problems [29]. Several studies have demonstrated that self-efficacy operates as a buffer of daily stress [30] and as a strong predictor of mental health [31]. For homosexuals, constant doubt and uncertainty about their abilities can be stressful and can contribute to rising levels of depression [32]. Homosexuals with low self-efficacy are less willing to seek medical treatment than those with high self-efficacy and have a higher risk of suicide [33]. Based on these findings, this study proposes Hypothesis 2: homosexuals’ self-efficacy could negatively predict their mental health status.

### 1.3. Mediating Role of Self-Efficacy

Social support comes from circumstances outside the self, whereas self-efficacy is part of the self. Self-efficacy reflects an individual’s subjective evaluation of his own ability, which can also be influenced by others, especially significant others. Especially in the context of Chinese culture, one’s self-appraisal is influenced by significant others [34]. The more social support an individual receives, the more encouragement and affirmation he receives, which further increases his self-efficacy [35]. A large number of studies have found that social support can enhance self-efficacy [36]. As mentioned above, perceived social support and self-efficacy can both influence mental health. Studies on different occupational groups found that perceived social support affects mental health through the mediating effect of self-efficacy [37,38]. For Chinese homosexuals, recent studies found that they perceived lower social support than heterosexual men [23] and had a lower level of self-efficacy than the general public [33]. Accordingly, this study proposes Hypothesis 3: self-efficacy has a mediating effect between perceived social support and anxiety and depression in homosexuals.

### 1.4. The Practical Significance of This Study

As mentioned above, homosexuals face different challenges compared to heterosexuals. In addition to external prejudice, discrimination and social exclusion, there are also internal self-identity, internalized homophobia and other mental health risks. In addition, the source and amount of social support that homosexuals receive are also different from those of heterosexuals, which is very important for them to cope with the pressure. The Minority Stress Model explains their internal mechanism of coping with stress, which is different from the stress-coping model of the general population [39]. Although numerous studies have explored the mental health of homosexuals, few have examined the protective factors of social support and self-efficacy among homosexuals in China. In the context of Chinese culture, homosexuals face different stressors from those in the West, and their ways of coping with stress are also different. How do they use external and their own coping resources to maintain mental health? The research on the protective factors of mental health and the internal mechanism of coping with stress in homosexuals is helpful to provide a professional reference for community public health service agencies.

According to the Minority Stress Model, community mental health service agencies can promote and guide the population to reduce discrimination and social exclusion of homosexuals to reduce the distal stressors, whereas targeted social support may minimize the proximal stressors of homosexuals. This may provide new ways for community public health agencies to work with community support and peer support to promote resilience and improve mental health for homosexuals.

### 1.5. Research Framework

The purpose of this study was to explore the roles of social support and self-efficacy in promoting mental health among Chinese homosexuals. Based on existing research literature and theoretical models, we propose three research hypotheses:

**Hypothesis** **1.** *Perceived social support of homosexuals negatively predicts mental health status with depression and anxiety as indicators*.

**Hypothesis** **2.** *Homosexuals’ self-efficacy negatively predicts their mental health status*.

**Hypothesis** **3.** *Self-efficacy has a mediating effect between perceived social support and anxiety and depression in homosexuals*.

**Figure 1:**
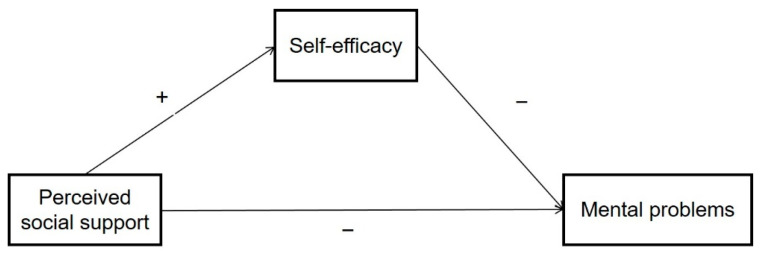
The correlation model among variables. Note: +: Positive correlation; −: Negative correlation.

Figure 1 shows the hypothetical relationships between these three variables.

## 2. Methods

### 2.1. Participants

Due to the dispersed and hidden nature of homosexuals, this study collected data from an online questionnaire survey. The gay dating software “Blued”, lesbian dating software “Rela”, Baidu post bar “Gay bar” and “Les bar” were used for publicity and questionnaire distribution. All questionnaires were completed through an online questionnaire website, “Star of questionnaire” (https://www.wjx.cn/, accessed on 5 September 2022). The purpose of the study and the investigation process were explained to the participants prior to the study. The survey was voluntary and anonymous. Written consent was obtained from all participants. This study was approved by the Academic Ethics Committee of Chongqing University of Arts and Science. The participants were asked to report their sexual orientation. A total of 209 participants reported being homosexuals, including 97 men and 112 women. Fifteen participants were removed from the sample due to missing data. Finally, the remaining 197 participants were included in the data analysis. The characteristics of the participants are shown in Table 1.

### 2.2. Measures

#### 2.2.1. Perceived Social Support Scale (PSSS)

Perceived social support was measured by the Perceived Social Support Scale (PSSS) [19]. The scale measures the level of support an individual perceives from family, friends and other people. There are 12 items in the Chinese revised scale [40], and the seven-point Likert score is adopted, scored from “1 (very disagreement)–7 (very agreement)”. Higher scores indicate higher perceived social support. The Cronbach’s coefficient of the total scale was 0.930 in the present study.

#### 2.2.2. General Self-Efficacy Scale (GSES)

Self-efficacy was measured by the General Self-efficacy Scale (GSES) [41]. The scale measures an individual’s overall level of confidence in dealing with challenges and difficulties in different contexts. The Chinese revised scale [42] has 10 items, and each item is rated on a 4-point scale ranging from 1 (Not at all true) to 4 (Exactly true). Higher scores indicate higher self-efficacy. The Cronbach’s coefficient of the total scale was 0.927 in the present study.

#### 2.2.3. Self-Rating Anxiety Scale (SAS)

Anxiety was measured by Self-rating Anxiety Scale (SAS) [43], and the Chinese revised scale [44] was adopted in the present study. Participants were asked to rate the frequency of anxiety symptoms described in the items in the last week. There are 20 items in this scale, and each item is rated on a 4-point scale ranging from 1 (Never or very little time) to 4 (Most or all of the time). The scores of the 20 items were summed to obtain the original score of the scale. Then, the original score was multiplied by 1.25, and the whole score was taken to get the standard score. Higher scores indicate higher levels of anxiety. The Cronbach’s coefficient of the total scale was 0.873 in this study.

#### 2.2.4. Self-Rating Depression Scale (SDS)

Depression was measured by the Self-rating Depression Scale (SDS) [45], a 20-item instrument. The Chinese revised scale [46] was adopted in this study. Participants were asked to rate the frequency of depressive symptoms in the last week on a Likert scale ranging from 1 (Never or very little time) to 4 (Most or all of the time). The standard score of the scale is calculated in the same way as SAS. Higher scores indicate higher levels of depression. The Cronbach’s coefficient of the total scale was 0.835 in this study. 

### 2.3. Statistical Analysis

The data were analyzed with the SPSS 21.0 software (SPSS Inc., Chicago, IL, USA). Correlation analysis and regression analysis were used to test the relationships among perceived social support, self-efficacy, anxiety and depression. SPSS Macro PROCESS (PROCESS 4.0 is written by Andrew F. Hayes, http://www.Afhayes.com, accessed on 20 September 2022) was used to test the mediating effect of self-efficacy on perceived social support, anxiety and depression of homosexuals.

## 3. Results

### 3.1. Common Method Bias

In order to minimize the common method bias, this study was conducted in strict accordance with the psychometric requirements. The survey was conducted anonymously at different times and on online platforms. Some items of the scales were scored in reverse. The Harman single-factor test was further used to detect the common method bias [47]. Results identify 22 factors with eigenvalues of greater than 1 and the first principal factor explained 22.38% of the variance (<40%), indicating the absence of any serious common method bias in the present study [48].

### 3.2. Descriptive and Correlation Analysis

According to the Chinese norm, the cutoff value of an SAS standard score is 50, where 50–59 is classified as mild anxiety, 60–69 as moderate anxiety, and 70 or more as severe anxiety [49]; the cutoff value of an SDS standard score is 53, where 53–62 is classified as mild depression, 63–72 as moderate depression, and ≥73 as severe depression [50]. In this study, participants with mild anxiety accounted for 25.9%, moderate anxiety for 14.2% and severe anxiety for 6.6%; 26.4% of the participants had mild depression, 23.4% had moderate depression and 4.1% had severe depression. 

Pearson correlation analysis was used to examine the relationships among perceived social support, self-efficacy, anxiety and depression. As shown in Table 2, the correlation analysis results showed that the total score of perceived social support and its dimensions were significantly correlated with self-efficacy, anxiety and depression. Specifically, the total score of perceived social support and self-efficacy were negatively correlated with anxiety and depression. These results verified Hypothesis 1 and Hypothesis 2.

### 3.3. The Mediating Effect of Self-Efficacy on Perceived Social Support and Anxiety and Depression

The macro program PROCESS of SPSS was used to test the mediation model, and model 4 in PROCESS was used for regression analysis. Regression analyses were conducted using perceived social support as the independent variable, self-efficacy as the mediating variable and anxiety and depression as the dependent variables, respectively. The results are shown in Table 3. Perceived social support could negatively predict anxiety (β = −0.30, t = −4.35, *p* < 0.001) and depression (β = −0.50, t = −8.02, *p* < 0.001). When the mediating variables were included, the direct predictive effects of perceived social support on anxiety (β = −0.27, t = −3.84, *p* < 0.001) and depression (β = −0.41, t = −6.72, *p* < 0.001) were still significant. Perceived social support had a significant positive predictive effect on self-efficacy (β = 0.29, t = 4.23, *p* < 0.001). Self-efficacy has a significant negative predictive effect on depression (β = −0.29, t = −4.66, *p* < 0.01) but not on anxiety (β = −0.08, t = −1.13, *p* > 0.05).

The non-parametric percentile bootstrap method with bias correction was used to test the mediating effect. The 95% confidence intervals were calculated by repeated sampling 5000 times, and statistical significance was indicated if the confidence intervals did not contain 0 [51]. In this study, when depression was the dependent variable, the upper and lower bounds of the bootstrap 95% confidence intervals for the direct effect of perceived social support and the mediating effect of self-efficacy did not include 0 (see Table 4). These results suggest that perceived social support not only directly predicts depression in homosexuals, but also predicts depression through the mediating effect of self-efficacy. The direct effect (−0.36) and mediating effect (−0.07) accounted for 83.25% and 16.75% of the total effect, respectively.

When anxiety was the dependent variable, the upper and lower limits of the bootstrap 95% confidence interval for the direct effect of perceived social support did not contain 0, but the upper and lower limits of the bootstrap 95% confidence interval for the mediated effect of self-efficacy did contain 0 (see Table 5). This suggests that perceived social support can only directly predict anxiety in homosexuals, and self-efficacy does not have a mediating effect between perceived social support and anxiety.

**Figure 2:**
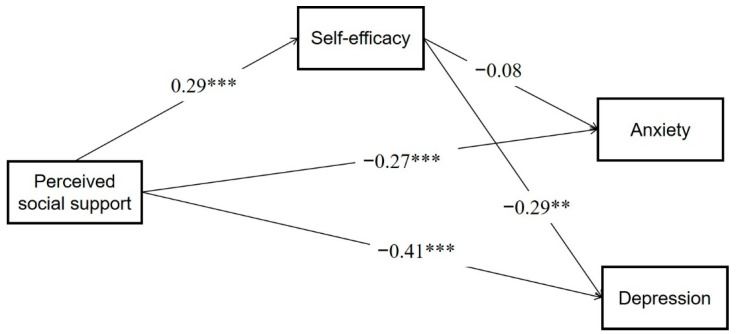
The mediating effects model for self-efficacy. Note: **: *p* < 0.01; ***: *p* < 0.001.

In summary, self-efficacy only mediates between perceived social support and depression, not between perceived social support and anxiety. Therefore, the results of this study partially validated Hypothesis 3 (See Figure 2).

## 4. Discussion

This study investigated the mental health of Chinese homosexuals and analyzed the relationships between perceived social support, self-efficacy, anxiety and depression. The mediating effect of self-efficacy on the relationship between perceived social support and anxiety and depression was also explored. The results of this study showed that 53.9% of the participants had depression symptoms (mild depression accounted for 26.4%, moderate depression for 23.4% and severe depression for 4.1%). In addition, 46.7% of the participants had anxiety problems (mild anxiety accounted for 25.9%, moderate anxiety accounted for 14.2% and severe anxiety accounted for 6.6%). The results of correlation analysis showed that perceived social support and self-efficacy were negatively correlated with anxiety and depression in homosexuals. The mediating effect analysis showed that self-efficacy had a mediating effect between perceived social support and depression but had no mediating effect between perceived social support and anxiety.

### 4.1. Mental Health of Homosexuals in China

This study found that depression and anxiety symptoms are prevalent among Chinese homosexuals, which is consistent with the results of previous studies [4,8]. A recent study using the Chinese version of SCL-90 measured anxiety and depression levels among Chinese gay men and found that the prevalence rates of depression, anxiety and comorbidity were 36.51%, 27.79% and 26.16%, respectively [6]. In addition, a study of Chinese lesbians found that 56.1% of lesbians had depressive symptoms [24]. These results suggest that despite differences in measurement instruments, Chinese homosexuals consistently report higher levels of depression and anxiety. As mentioned earlier, in addition to the general pressures of prejudice, discrimination, stigma or social exclusion [5,12], Chinese homosexuals also have to deal with the specific pressures of traditional Confucian values of filial piety [13,14]. Homosexual marriage is illegal in China, which means that homosexuals cannot pass on their heritage and continue the bloodline. This is in conflict with the value of filial piety. As a result, homosexuals in China need to cope with different stressors and face more mental health risks.

### 4.2. The Relationships among Perceived Social Support, Self-Efficacy and Mental Health

In this study, perceived social support and self-efficacy were significantly negatively correlated with anxiety and depression in homosexuals, which verifies hypotheses 1–2 and previous studies [20,26]. These results suggest that perceived social support and self-efficacy are both important protective factors affecting mental health of homosexuals. In Chinese society, good interpersonal relationships are very important to maintain mental health. The quality of social support an individual receives from others reflects the state of his interpersonal relationships. The research on Chinese sexual minority men has found that they received lower overall social support than Chinese heterosexual men from family, friends and significant others [23]. Notably, Chinese sexual minority men received less family support than support from friends and significant others, whereas heterosexuals received more family support than other kinds of support. Reduced family support can lead to more negative attitudes towards their sexuality [52], which can ultimately lead to difficulties in relationships [53] and increased mental health problems for homosexuals. This further validates the main effect model of social support.

Some studies regard self-efficacy as a core prevention criterion of mental health [54,55]. Like social support, self-efficacy is seen as a stress buffer [30]. As for the internal mechanism, some researchers have pointed out that individuals with high general self-efficacy are more likely to have lower risk perception, adopt more active coping strategies, and subsequently experience fewer mental health problems [31]. Compared to the general public, Chinese homosexuals have a lower sense of self-efficacy [33]. These results confirm the positive effect of self-efficacy on mental health in homosexuals.

### 4.3. Mediating Effects of Self-Efficacy

This study found that perceived social support not only has a direct effect on the depression status of homosexuals, but also indirectly affects the depression level by improving self-efficacy. According to the stress-coping model, social support and self-efficacy are two important stress-coping resources, which play an important role in maintaining mental health [56]. The various social supports obtained from the outside world will be internalized into self-cognition and self-concept, which will further affect mental health. Especially in the Chinese cultural context, receiving social support from others means receiving affirmation and encouragement from others, which in itself is a recognition of one’s ability [35]. This positive behavioral feedback will further increase the individual’s self-efficacy. Self-efficacy, on the other hand, can improve individuals’ cognitive evaluation of stressors during stress coping, which can help them to adopt a more positive coping style and enhance the efficiency of problem-solving [56]. Thus, receiving more social support increases self-efficacy, improves stress coping, and produces a more positive self-evaluation, which in turn reduces the occurrence of depression. In the face of different stressors, a large number of studies have confirmed that self-efficacy plays a mediating role in social support and mental health [37,38,57].

It is worth noting that self-efficacy did not mediate the relationship between perceived social support and the anxiety of homosexuality. As mentioned above, social support and self-efficacy have direct positive effects on mental health. According to the minority stress theory, in addition to general pressures, homosexuals also have to deal with distal pressures such as prejudice, discrimination and social exclusion, and proximal pressures such as fear of rejection and internalized homophobia [3]. When homosexuals receive more social support, this will offset the impact of distal pressures to some extent, especially in a collectivist culture where social support means that others stand with them [58]. This, to some extent, reduces the psychological threat of the external environment to homosexuals. Furthermore, higher self-efficacy can further reduce the psychological threat of external stressors to individuals. Thus, homosexuals who receive social support from others or have high self-efficacy can directly reduce anxiety levels. The role of social support here validates the main effect model.

### 4.4. Limitations

In this study, data were collected online and participants participated in the questionnaire survey voluntarily. The researchers were unable to control the questionnaire survey process on site to make sure that everyone answered carefully and according to their real thoughts. On the other hand, it is difficult to achieve random sampling in the study of homosexuals, which limits the representativeness of the sample. Future studies should further increase the sample size and the channels for recruiting subjects to further improve the representativeness of the sample.

In addition, the mental health conditions in this study are represented primarily by anxiety and depression. However, in real life, homosexuals face stress that brings them much more than anxiety and depression. On the other hand, stress does not always have a negative impact on homosexuals. Researchers have published findings about the co-occurrence of positive affect with negative affect during chronic stress and the adaptive functions of positive affect during chronic stress [59]. Future research should adopt both positive and negative indicators to measure the mental health status of homosexuals.

Finally, this study focuses on homosexuals, who are a subtype of sexual minorities (including gay, lesbian, bisexual and transgender). Different subtypes have different mental health characteristics. For example, researchers have found that bisexuals face higher mental health risks than homosexuals [60]. This suggests that research on the mental health of other types of sexual minorities, such as bisexuals and transgenders, is also necessary. In addition, the addition of a heterosexual control group in future studies could further help us understand the applicability of different theoretical models of mental health.

## 5. Conclusions

Despite some limitations, this study confirms the positive effects of perceived social support and self-efficacy for homosexuals. In conclusion, Chinese homosexuals have some anxiety and depression symptoms. Perceived social support and self-efficacy of homosexuals negatively predict anxiety and depression. Self-efficacy mediates the relationship between perceived social support and depression.

These findings help us to understand the roles of protective factors in homosexuals’ mental health in China and have certain implications for the practice of mental health services in community public health agencies. These agencies can carry out extensive publicity of gender knowledge popularization and guide the public to eliminate discrimination and stigma against homosexuals, which is conducive to eliminating external pressure on homosexuals. Organizing support groups for homosexuals and providing peer social support can increase their self-identity, improve their sense of self-efficacy, and help them cope with stress more effectively. Future research could explore the effects of these interventions.

## Figures and Tables

**Table 1:** Characteristics of the participants.

	Categories	Numbers	Percentage
Residence	City	166	84.2%
	Rural area	31	15.8%
Gender	Male	88	44.7%
	Female	109	55.3%
Age	<18	24	12.2%
	18–25	126	64%
	26–45	43	21.8%
	>45	4	2%
Education level	≤Junior high school	13	6.6%
	High school	34	17.3%
	Undergraduate	146	74.1%
	≥Master	4	2%

**Table 2:** Descriptive statistics and correlation analysis results.

	M	SD	1	2	3	4	5	6	7
1 Family support	18.12	6.17	1						
2 Friends support	20.7	4.94	0.523 **	1					
3 Other support	21.07	4.96	0.606 **	0.776 **	1				
4 Perceived social support	59.89	13.93	0.844 **	0.863 **	0.900 **	1			
5 self-efficacy	25.15	6.63	0.344 **	0.146 *	0.241 **	0.290 **	1		
6 Anxiety	49.47	12.33	−0.208 **	−0.295 **	−0.283 **	−0.297 **	−0.160 *	1	
7 Depression	53.02	12.02	−0.417 **	−0.415 **	−0.466 **	−0.498 **	−0.408 **	0.708 **	1

Note: M = mean; SD = standard deviation; *: *p* < 0.05; **: *p* < 0.01.

**Table 3:** Mediation model test of self-efficacy.

Regression Equation (N = 197)		Fitting Indexes			Significance of Coefficient	
Outcome Variables	Predict Variables	R	R²	F(df)	β	t
Depression	Perceived social support	0.50	0.25	64.25(1) ***	−0.50	−8.02 ***
Anxiety	Perceived social support	0.30	0.09	18.92(1) ***	−0.30	−4.35 ***
Self-efficacy	Perceived social support	0.29	0.08	17.91(1) ***	0.29	4.23 ***
Depression	Self-efficacy	0.57	0.32	46.40(2) ***	−0.29	−4.66 ***
	Perceived social support				−0.41	−6.72 ***
Anxiety	Self-efficacy	0.31	0.09	10.11(2) ***	−0.08	−1.13
	Perceived social support				−0.27	−3.84 **

Note: **: *p* < 0.01; ***: *p* < 0.001.

**Table 4:** Total effect, direct effect and mediating effect (depression as dependent variable).

	Effect Size	Boot SE	Boot CL Lower	Boot CL Upper	Relative Effect Value
Total effect	−0.43	0.05	−0.54	−0.33	
Direct effect	−0.36	0.05	−0.45	−0.26	83.25%
Mediating effect	−0.07	0.03	−0.15	−0.02	16.75%

**Table 5:** Total effect, direct effect and mediating effect (anxiety as dependent variable).

	Effect Size	Boot SE	Boot CL Lower	Boot CL Upper	Relative Effect Value
Total effect	−0.26	0.07	−0.40	−0.12	
Direct effect	−0.24	0.08	−0.38	−0.08	92.14%
Mediating effect	−0.02	0.03	−0.09	0.03	7.86%

## Data Availability

The datasets used and analyzed in the study are available from the corresponding author on request.
